# Supplementary material for: Trypanosoma cruzi DNA Identification in Breast Milk from Mexican Women with Chagas Disease
Source: Microorganisms. 2024 Dec 21;12(12):2660. doi: 10.3390/microorganisms12122660 (PMC11728801; doi:10.3390/microorganisms12122660)
Supplement: Supplementary file 1 [file microorganisms-12-02660-s001.zip › microorganisms-3327368-supplementary.pdf]

Trypanosoma cruzi DNA identification in breast milk from Mexican women with Chagas disease

María del Pilar Crisóstomo-Vázquez[1], Griselda Rodríguez-Martínez[2], Verónica Jiménez-Rojas[2], Leticia Eligio-García[2], Alfonso Reyes-López[3], María Hernández-Ramírez[4], Francisco Hernández-Juárez[5], José Luis Romero-Zamora[6], Silvia Guadalupe Vivanco-Tellez[7], Fortino Solorsano-Santos[7], Víctor M. Luna-Pineda[2,*], and Guillermina Campos-Valdez[2,*]

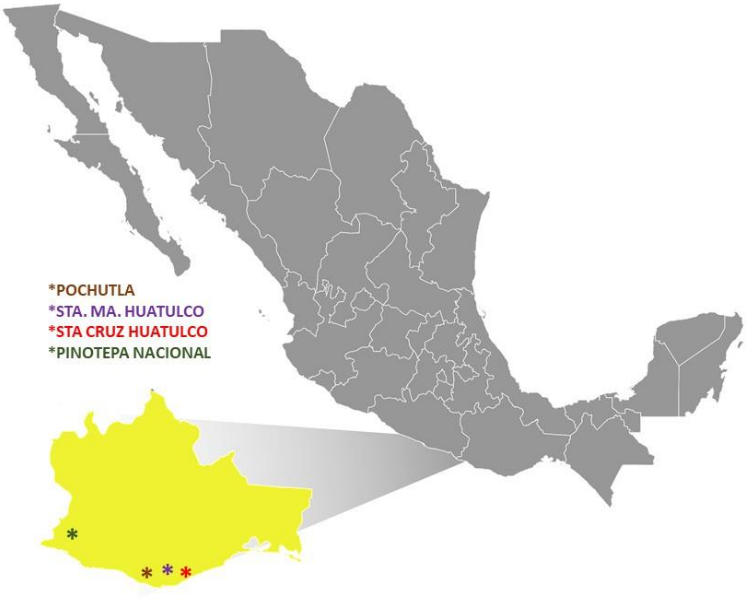

Supplementary Figure S1. Localisation of four regional hospitals in the state of Oaxaca. These hospitals are located on México´s Pacific coast: Sta. Ma. Huatulco, Sta. Cruz Huatulco and Sn. Pedro Pochutla (North 15° 45′ 17.39″ latitude, West 096° 7′ 52.29″ longitude), Pinotepa Nacional is North 16° 20´ 17´´ latitude, West 98° 03´ 01´´ longitude.
